# (*S*)-3-hydroxyacyl-CoA dehydrogenase/enoyl-CoA hydratase (FadB’) from fatty acid degradation operon of *Ralstonia eutropha* H16

**DOI:** 10.1186/s13568-014-0069-0

**Published:** 2014-08-28

**Authors:** Elena Volodina, Alexander Steinbüchel

**Affiliations:** 1 Institut für Molekulare Mikrobiologie und Biotechnologie, Westfälische Wilhelms-Universität Münster, Corrensstraße 3, Münster, D-48149, Germany; 2 King Abdulaziz University, Jeddah, Saudi Arabia

**Keywords:** Fatty acid metabolism, 3-hydroxyacyl-CoA dehydrogenase/enoyl-CoA hydratase, Ralstonia eutropha H16

## Abstract

In this study (*S*)-3-hydroxyacyl-CoA dehydrogenase/enoyl-CoA hydratase (H16_A0461/FadB’, gene ID: 4247876) from one of two active fatty acid degradation operons of *Ralstonia eutropha* H16 has been heterologously expressed in *Escherichia coli*, purified as protein possessing a His-Tag and initially characterized. FadB’ is an enzyme with two catalytic domains exhibiting a single monomeric structure and possessing a molecular weight of 86 kDa. The C-terminal part of the enzyme harbors enoyl-CoA hydratase activity and is able to convert *trans*-crotonyl-CoA to 3-hydroxybutyryl-CoA. The N-terminal part of FadB’ comprises an NAD^+^ binding site and is responsible for 3-hydroxyacyl-CoA dehydrogenase activity converting (*S*)-3-hydroxybutyryl-CoA to acetoacetyl-CoA. Enoyl-CoA hydratase activity was detected spectrophotometrically with *trans*-crotonyl-CoA. (*S*)-3-Hydroxyacyl-CoA dehydrogenase activity was measured in both directions with acetoacetyl-CoA and 3-hydroxybutyryl-CoA. FadB’ was found to be strictly stereospecific to (*S*)-3-hydroxybutyryl-CoA and to prefer NAD^+^. The *K*_*m*_ value for acetoacetyl-CoA was 48 μM and *V*_*max*_ 149 μmol mg^−1^ min^−1^. NADP(H) was utilized at a rate of less than 10% in comparison to activity with NAD(H). FadB’ exhibited optimal activity at pH 6–7 and the activity decreased at alkaline and acidic pH values. Acetyl-CoA, propionyl-CoA and CoA were found to have an inhibitory effect on FadB’. This study is a first report on biochemical properties of purified (*S*)-stereospecific 3-hydroxyacyl-CoA dehydrogenase/enoyl-CoA hydratase with the inverted domain order from *R. eutropha* H16. In addition to fundamental information about FadB’ and fatty acid metabolism, FadB’ might be also interesting for biotechnological applications.

## Introduction

*Ralstonia eutropha* H16 is a Gram-negative β-proteobacterium. Recently, this bacterium was reclassified to *Cupriavidus necator* H16 (Vandamme and Coenye [2004]); however, *R. eutropha* H16 is the most used designation in the recent scientific literature. *R. eutropha* is able to utilize various fatty acids (FAs) as sole carbon source and to synthesize poly(3-hydroxybutyrate) (PHB) up to 80% or even more of its dry cell weight (Anderson and Dawes [1990]). Two (*S*)-3-hydroxyacyl-CoA dehydrogenase/enoyl-CoA hydratases, H16_A0461/FadB’ and H16_A1526/FadB1, are involved in the FA degradation in *R. eutropha* H16 (Brigham et al. [2010]). According to their annotation, FadB’ and FadB1 possess an enoyl-CoA hydratase activity, catalyzing hydrogenation of the unsaturated enoyl Coenzyme A (CoA); and a 3-hydroxyacyl-CoA dehydrogenase activity, i.e. oxidation of the hydroxyl group into a keto group using one NAD^+^ molecule.

The genome sequence of *R. eutropha* H16 unravelled a variety of genes and operons, which could be responsible for the degradation of FAs in this organism (Pohlmann et al. [2006]; Reinecke and Steinbüchel [2009]). However, little is still known about how the fatty acid metabolism of *R. eutropha*. It is assumed that β-oxidation in *R. eutropha* H16 is similar to the well-studied pathway of *Escherichia coli* (Binstock and Schulz [1981]; Black and DiRusso [1994]; Clark and Cronan [1996]; Riedel et al. [2014]). *E. coli* contains two *f*atty *a*cid *d*egradation (*fad*) operons. Their expression is induced when cells are grown in presence of FAs containing 12 or more carbon atoms (Klein et al. [1971]). Recently, Brigham et al. ([2010]) showed that two *fad* operons (H16_A0459-A0464 and H16_A1526-A1531) were also up-regulated when *R. eutropha* was grown in presence of trioleate. However, their moderate expression is also observed in the absence of FAs (Schimizu et al. [2013]). Initially, FAs are activated to acyl-CoA by an ATP-depended acyl-CoA synthase (FadD) resulting in an acyl-CoA (Black et al. [1992]). Then an acyl-CoA dehydrogenase (FadE) introduces a double bound between the FA’s 2nd and the 3rd carbon atoms yielding *trans*-2-enoyl-CoA. Next, two reactions catalyzed by FadB and yielding 3-ketoacyl-CoA (see above) are following. FadB_*Ec*_ is a single trifunctional fused polypeptide exhibiting enoyl-CoA hydratase (EC 4.2.1.17), epimerase (EC 5.1.2.3) and isomerase/3-hydroxyacyl-CoA dehydrogenase (EC 1.1.1.35; EC 5.3.3.8) activities. FadB_*Ec*_ forms, together with a thiolase (FadA), a multienzyme complex (Black and DiRusso [1994]; Pramanik et al. [1979]; Pawar and Schulz [1981]; Yang et al. [1988]). The final step of the FA degradation cycle, catalyzed by FadA, is a cleavage of 3-ketoacyl-CoA to an acetyl-CoA and an acyl-CoA molecule shortened for two carbon atoms. The shortened acyl-CoA enters again the β-oxidation, whereas acetyl-CoA enters the Krebs cycle (Black and DiRusso [1994]; Clark and Cronan [1996]) or, as it is shown for *R. eutropha*, the PHB synthesis.

Synthesis of PHB in *R. eutropha* comprises three steps. The first two steps of this pathway are similar with the last reactions of the β-oxidation, though in a reverse direction. A condensation of two acetyl-CoA molecules by a thiolase (PhaA) to acetoacetyl-CoA (Haywood et al. [1988a]; Oeding and Schlegel [1973]) and a reduction of acetoacetyl-CoA to (*R*)-3-hydroxybutyryl-CoA by a NADPH-dependent acetoacetyl-CoA reductase, PhaB (EC.1.1.1.36; Haywood et al. [1988a]). The third step of PHB synthesis is a stereospecific polymerization of (*R*)-3-hydroxybutyrate (3HB) by PHB synthase (PhaC) (Peoples and Sinskey [1989]; Schubert et al. [1991]). Beside (*R*)-3HB, (*R*)-3-hydroxyvalerate and (*R*)-3-hydroxyhexanoate can be incorporated into polyhydroxyalkanoates (PHAs) (Dennis et al. [1998]; Slater et al. [1998]).

Thus, 3-ketoacyl-CoAs and 3-hydroxyacyl-CoAs are the intermediates of either β-oxidation or PHA metabolism. Hence, (*S*)-3-hydroxyacyl-CoA dehydrogenase/enoyl-CoA hydratases, beside their fundamental role in FA degradation (Brigham et al. [2010]), might mediate between FA metabolism and PHA synthesis. The studies on a *fadB*’ and *fadB1* deletion mutants of *R. eutropha* revealed a special role of FadB’ in provision of precursors for PHA synthesis (Insomphun et al. [2014]). The *fadB*’ deletion led to the reduction of the 3HB content in the copolymer on FAs as sole carbon source. However, if only FadB’ was active in the *fadB1* deletion mutant grown on fatty acids, the 3HB content was similar with the wild type. On the other hand, the *fadB1* deletion mutant exhibited an increased 3-hydroxyhexanoate (3HHx) fraction in the copolymer, compared to the wild type and *fadB*’ gene deletion mutant (Insomphun et al. [2014]). The different role of FadB’, in comparison to FadB1, in the PHA precursor supply is not clear. Moreover, FadB’ exhibits a different structure as its isoenzyme FadB1 and well studied enzyme of *E. coli* (Insomphun et al. [2014]). FadB’ is a polypeptide comprising a N-terminal 3HCDH_N-domain, responsible for NAD binding, and a C-terminal enoyl-CoA hydratase domain and little is known about the enzymatic properties of the novel type of enzyme with the inverted domain order.

Amongst the provision of the intermediates for the PHA synthesis, a different field where FadB’ could be exploited is the production of enantiomeric pure chemicals, as it was shown for (*S*)- or (*R*)-3-hydroxybutyrate in *E. coli* (Lee et al. [2008]; Tseng et al. [2009]). Another example is an engineered microbial pathway for synthesis of linear alcohols, which is based on β-oxidation and requires both 3-hydroxyacyl-CoA dehydrogenase and enoyl-CoA hydratase activities (Machado et al. [2012]). For this purpose another 3-hydroxyacyl-CoA dehydrogenase (PaaH1) from *R. eutropha* and a crotonase were studied intensively. PaaH1 was heterlogously expressed and crystallized (Machado et al. [2012]; Kim et al. [2014]). At last, since the purified FadB’ is stable over a relatively long time period, its *in vitro* use for coupled enzyme assays may have a potential application (Haywood et al. [1988a]; Volodina et al. [2014]). Here, we report about the purification and initial characterization of a heterologously expressed His-tagged H16_A0461/FadB’ (Gene ID: 4247876) from one of the active *fad* operons of *R. eutropha*.

## Materials and methods

### Bacterial strains, oligonucleotides and plasmids

Bacterial strains, oligonucleotides and plasmids used in this study are listed in the Table 1. *Escherichia coli* BL21 was used for inducible expression of the enzymes of interest.

**Table 1:** Bacterial strains, plasmids and oligonucleotides used in this study

	**Description**	**Reference or source**
*Strains*		
*Ralstonia eutropha* H16	Wild type	DSM 428
*Escherichia coli*		
BL21 (DE3)	F^−^*omp*T *hsd*S_B_ (r_B_^−^, m_B_^−^) *gal dcm* (DE3)	Novagen
*Plasmids*		
pCOLADuet-1::*3HAD*	pCOLADuet-1 with *fadB’* as *Bam*HI/*Hin*dIII fragment	Volodina et al. [2014]
pET-19b::*pct*	pET-19b with *pct* as *Nde*I/*Xho*I fragment	Lindenkamp et al. [2013]
pET-23a	*E. coli* expression vector (Ap^r^, T7 promoter)	Novagen
pET-23a::*bktB*	pET-23a with *bktB* as *Nde*I/*Hin*dIII fragment	This study
*Oligonucleotides*		
Forward primer BktB *Nde*I*	AAA*CATATG*ACGCGTGAAGTGGTAGTGGTAAGC	This study
Reverse primer BktB *Hind*III*	AAA*AAGCTT*GATACGCTCGAAGATGGCGGC	This study

^*^Restriction sites are highlighted in italics.

### Chemicals and materials

*Trans*-crotonyl-CoA, (*R*)-3-hydroxybutyrate, (*S*)-3-hydroxybutyric acid, lactate dehydrogenase were purchased from Sigma-Aldrich (Steinheim, Germany). Coenzyme A, NAD^+^, NADH, NADP^+^ and NADPH were available from Gerbu Biochemicals GmbH (Gaiberg, Germany). Acetic acid anhydride was form Acros Organics (Geel, Belgium) and propionic and crotonic acid anhydrides were from Merck Millipore (Darmstadt, Germany) and His SpinTrap™ chelating metal affinity columns were purchased from GE Healthcare (Munich, Germany).

### Synthesis of acyl-CoA thioesters

Acetyl-CoA, propionyl-CoA and crotonyl-CoA were prepared chemically from corresponding anhydrides and CoA according to the method of Simon and Shemin ([1953]). (*S*)-3-hydroxybutyryl-CoA was synthesized enzymatically by propionate-CoA transferase for each assay *in situ* as described below.

### DNA isolation and recombinant DNA techniques

Chromosomal DNA of *R. eutropha* H16 was isolated according to the method of Marmur ([1961]). Plasmid DNA was isolated by using the peqGOLD Plasmid Miniprep Kit (Peqlab) according to the manufacturer’s manual. PCRs were carried out in an Omnigene HBTR3CM DNA thermal cycler (Hybaid, Heidelberg Germany) using genomic DNA of *R. eutropha* H16 as a template, oligonucleotides (Table 1) and *Taq*-DNA polymerase according to the manufacturer’s instructions (Thermo Scientific, Dreieich, Germany). The oligonucleotide annealing temperature was 56°C, and the elongation time at 72°C was 2.5 min. PCR products were isolated from an agarose gel and were purified by using a peqGOLD Gel Extraction Kit (Peqlab) according to the manufacturer’s instructions. T4 DNA Ligase was purchased from Thermo Scientific (Dreieich, Germany). Oligonucleotides were synthesized by Eurofins MWG (Ebersberg, Germany). Sequencing reactions of DNA fragments were carried out according to a standard procedure at the Sequence Laboratories Göttingen GmbH (Göttingen, Germany). Competent cells of *E. coli* were prepared and transformed with plasmids by the CaCl_2_ procedure as described by Hanahan (Hanahan [1983]).

### Sequence data analysis

The nucleotide and amino acid sequences were extracted from the National Center for Biotechnology Information (NCBI) nucleotide sequence and amino acid database (http://www.ncbi.nlm.nih.gov/Genbank/index.html). Gene accession numbers: *bktB* (AM260479) and *fadB*’ (AM260479). For determination of amino acid identity the program BlastP from the National Center for Biotechnology Information (http://www.ncbi.nlm.nih.gov/) was used (Altschul et al. [1997]). Phylogenetic tree and amino acid sequence alignment were generated using CLUSTAL X program with PhaA as a reference (Thompson et al. [1997]).

### Heterologous expression and purification of FadB’ and coupling enzymes

To obtain overexpressed enzymes, strains of *E. coli* BL21 harboring pCOLADuet-1::*3HAD* (Volodina et al. [2014])*,* pET19b::*pct* (Lindenkamp et al. [2013]) or pET23a::*bktB* were used. β-Ketothiolase (BktB) and propionate CoA-transferase (Pct) (Lindenkamp et al. [2013]) from *R. eutropha* H16 were used as auxiliary enzymes for the enzyme assays. FadB’, Pct and β-ketothiolase were heterologously expressed in *E. coli* BL21. Isopropyl-β-D-1-thiogalactopyranoside (IPTG) was used for induction of the expression (0.5 mM IPTG, 30°C). Purification of the enzymes was carried out with a His SpinTrap™ column according to the manufacturer’s instructions via N-terminal His-tag and stored as described before (Lindenkamp et al. [2013]; Volodina et al. [2014]). Binding buffer (100 mM Tris–HCl, 500 mM NaCl, 20 mM imidazole, pH 7.5) was used for cells resuspension and His SpinTrap™ column equilibration. The bound enzyme was washed with 3 volumes of 100 mM Tris–HCl containing 500 mM NaCl plus 100 mM imidazole (pH 7.5) and was then eluted with the same buffer containing 500 mM imidazole (Volodina et al. [2014]). FadB’ was stabilized by addition of glycerol to a final concentration of 50% (v/v) and was then stored at −20°C for over one month without loss of activity.

The purity of enzymes was determined by sodium dodecyl sulfate (SDS) polyacrylamide gel electrophoresis (PAGE) (Suppl. 3; Laemmli [1970]). Protein concentrations were measured by using the method of Bradford (Bradford [1976]). The protein samples (5–10 μg protein) were resuspended in gel loading buffer (0.6% (w/v) SDS, 1.25% (v/v) β-mercaptoethanol, 0.25 mmol l^−1^ EDTA, 10% (v/v) glycerol, 0.001% (w/v) bromophenol blue, 12.5 mM Tris–HCl [pH 6.8]) and were separated in 12.5% (w/v) SDS-polyacrylamide gels as described by Laemmli ([1970]). The proteins were stained with Coomassie brilliant blue R-250 (Weber and Osborn [1969]).

### Determination of the molecular mass

The molecular mass of FadB’ was determined on a Superdex 200 HR 10/30 column (Amersham Pharmacia Biotech) at a flow rate of 1 ml/min in 50 mM sodium phosphate buffer (pH7.4) containing 150 mM NaCl. Molecular mass standards used for calibration were from the Gel Filtration Calibration Kit HMW (GE Healthcare, UK).

### High performance liquid chromatography/mass spectrometry (HPLC/MS) assay

The purity of the synthesized CoA-thioesters and enzyme reaction products was determined with reverse phase high performance liquid chromatography-mass spectrometry (RP-HPLC-MS) by employing an UltiMate® 3000 HPLC apparatus (Dionex GmbH, Idstein, Germany) connected directly to an LXQ™ Finnigan™ (ThermoScientific, Dreieich, Germany) mass spectrometer. An Acclaim 120 C18 Reversed-Phase LC Column (4.6 × 250 mm, 5 μm, 120 Å pores; Dionex GmbH) was used at 30°C based on a method described earlier with slight modifications (Lindenkamp et al. [2013]; Schürmann et al. [2011]). A gradient system was used, with 50 mM ammonium acetate, pH 5.0 adjusted with acetic acid (A), and 100 % (v/v) methanol (B) as eluents. Elution occurred at a flow rate of 0.5 ml/min. Ramping was performed as follows: equilibration with 90 % A for 2 min before injection and afterwards a change from 90% to 10% eluent A in 25 min, followed by holding for 10 min and then returning to 90% eluent A within 10 min. CoA-thioesters were detected at 259 nm by a photodiode array detector. Tuning of the instrument was done by direct infusion of a solution of 0.4 mM CoA as shown before (Schürmann et al. [2011]).

### Enzyme assays

All the enzyme activities were assayed by continuous spectrophotometric assays as described below. The substrates were prepared fresh directly before each enzyme assay and the coupling enzymes were applied in purified form. All values are presented as mean values of at least two measurements with standard deviations.

*(i) Enoyl-CoA hydratase activity* of the FadB’ was measured with *trans*-2-crotonyl-CoA (Sigma-Aldrich, Germany) as a substrate. The reaction mixture contained 0.1 mM *trans*-crotonyl-CoA, 1.5 mM NAD^+^, 0.3 μg FadB’_*Re*_, 1 μg β-ketothiolase, 0.1 mM CoA in 100 mM Tris–HCl buffer, pH 7. The increase of absorption was monitored at 340 nm and 30°C (Binstock and Schulz [1981]).

For determination of inhibition of enoyl-CoA hydratase activity of FadB’ 0.1 mM crotonyl-CoA was prepared from crotonic acid anhydride and CoA. NAD^+^ regeneration with lactate dehydrogenase was applied to ensure the processing of the reaction in desired direction as described before (Haywood et al. [1988a]; Volodina et al. [2014]). The reaction mixture contained 100 mM Tris–HCl (pH 8.1), 0.1 mM crotonyl-CoA, 2 mM pyruvate, lactate dehydrogenase (9 U), 1.5 mM NAD^+^, 25 mM MgCl_2_ and different concentrations of CoA, acetyl-CoA or propionyl-CoA (0.1 mM, 0.5 mM or 1 mM) were additionally applied. The reaction was started by addition of 0.3 μg of FadB’ and the formation of acetoacetyl-CoA-Mg^2+^ (ε_303_ = 16500 M^−1^cm^−1^) complexes was monitored for 2 min at 303 nm at 30°C. One unit of enzyme activity was defined as the formation of 1 μmol acetoacetyl-CoA per min.

*(ii) 3-Hydroxyacyl-CoA dehydrogenase activity* of FadB’ was measured at 340 nm and 30°C with acetoacetyl-CoA in direction of 3-hydroxybutyryl-CoA formation. The reaction mixture contained 0.1 mM acetoacetyl-CoA, 0.2 mM NADH, 0.3 μg FadB’_*Re*_ in 100 mM Tris–HCl buffer, pH 7 (Haywood et al. [1988b]). For determination of inhibition of the 3-hydroxyacyl-CoA dehydrogenase activity of FadB’ by the potential inhibitors CoA or acetyl-CoA were added at a final concentration of 0.1 mM, 0.5mM or 1 mM. The kinetic parameters K_m_ and V_max_ for acetoacetyl-CoA were ascertained by varying its concentration (0.01 mM, 0.05 mM, 0.1 mM, 0.3 mM, 0.5 mM and 0.75 mM) in presence and absence of 0.1 mM acetyl-CoA and fitting the data to the Michaelis–Menten equation. Temperature optimum was determined by varying the temperature: 25°C, 30°C, 35°C, 40°C, 45°C, and 50°C. For determination of optimal pH at room temperature 100 mM Tris–HCl buffer at a range of 6–10 was applied, whereas the range 4–6 was measured in 10 mM acetic acid/acetate buffer.

*(iii) Stereospecificity of 3-hydroxybutyryl-CoA dehydrogenase* was determined by another coupled enzyme assay. Here, the ability of Pct to synthesize 3-hydroxybutyryl-CoA from acetyl-CoA and 3-hydroxybutyrate was used for *in situ* substrate synthesis as described before (Haywood et al. [1988a]; Volodina et al. [2014]). The reaction mixture contained: 100 mM Tris–HCl (pH 8.1), 0.1 mM acetyl-CoA, 25 mM (*S*)- or (*R*)-3-hydroxybutyric acid, 2 mM pyruvate, lactate dehydrogenase (9 U), 1.5 mM NAD^+^, Pct (10 μg) and 25 mM MgCl_2_. After pre-incubation of the enzyme mixture for 10 min at 30°C the reaction was started with addition of 0.3 μg of purified FadB’. The generated 3-hydroxybutyryl-CoA was converted to acetoacetyl-CoA by FadB’, and the formation of acetoacetyl-CoA-Mg^2+^ (ε_303_ = 16,500 M^−1^cm^−1^) complexes was monitored for 2 min at 303 nm at 30°C. One unit of enzyme activity was defined as the formation of 1 μmol acetoacetyl-CoA per min.

*(iv) NAD(H) and NADP(H) cofactor utilization* capability of FadB’ was ascertained in two directions. First, acetoacetyl-CoA was applied as a substrate for the enzyme assay, and the oxidation of 0.1 mM NADH or 0.1 mM NADPH was measured at 340 nm as described above. Second, (*S*)-3-hydroxybutyryl-CoA was synthesized by Pct as described for 3-hydroxybutyryl-CoA dehydrogenase assay, however, BktB was applied as coupling enzyme, and the assay mixture contained 0.1 mM acetyl-CoA, 25 mM 3-hydroxybutyrate, 10 μg Pct, 0.1 mM CoA, 1 μg BktB, 0.3 μg FadB. The reaction mixture was pre-incubated for 10 min at 30°C, and the change of absorption at 340 nm was measured for 5 minutes after addition of NAD^+^ or NADP^+^ to the final concentration 1 mM.

## Results

### *In silico* assay

**Figure 1:**
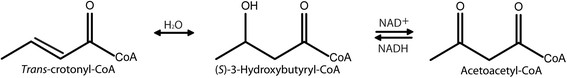
Enoyl-CoA hydratase and (S)-3-hydroxyacyl-CoA dehydrogenase reactions of FA degradation pathway, catalyzed by FadB’.

**Figure 2:**
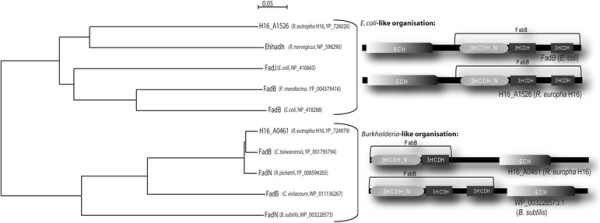
**Neighbor-joining phylogenetic tree (Clustal X; Thompson et al.**[1997]**) based on amino acid sequence of different 3-hydroxyacyl-CoA dehydrogenases and the organization of FadB enzymes with different domain order (modified from Insomphun et al.**[2014]**): I- enzymes with*E. coli-*like structure; II- enzymes with*Burkholderia*-like structure.** Accession numbers are given at the braces. Bar, 0.05 amino acid substitution per site.

FadB’ consists of 806 amino acids (AA) with a theoretical molecular weight of 85.9 kDa and a pI of 7.66. The C terminus is annotated as a crotonase-like family/enoyl-CoA hydratase (ECH) domain and reveals similarity to the N-terminus of FadB1 (34% identity), a variety of enoyl-CoA hydratases like H16_B0389 (261 AA, 34% identity) and H16_B0987 (255 AA, 30% identity) from *R. eutropha* H16 and other organisms. The enoyl-CoA hydratase reaction is shown in Figure 1. The N-terminus of FadB’_*Re*_ reveals similarities to genes of other 3-hydroxyacyl-CoA dehydrogenases of *R. eutropha* H16 like H16_A1102 (507 AA, 31% identity), H16_A0282 (284 AA, 33% identity), FadB1 (693 AA, 31% identity), and H16_B1652 (322 AA, 30% identity). This terminus comprises NAD binding (3HCDH_N) and 3HCDH domains and catalyzes the second step of the reaction shown in Figure 1. FadB1 is analogous to FadB’, although it possesses a reverse domain order (Insomphun et al. [2014]). Interestingly, a separate enoyl-CoA hydratase (H16_A0464, 11% identity) is located downstream of the H16_A0459-A0464 operon and reveals no significant similarity to the ECH-domain of FadB’_*Re*_. Amongst other bacteria most homologous to FadB’_*Re*_ enzymes were found in many of species of the genus *Burkholderia* and other bacteria. Similarly organized 3-hydroxyacyl-CoA dehydrogenases were also found in *Bacillus subtilis* and *Chromobacterium violaceum* (Figure 2). According to the amino acid sequence and the order of domains two different groups of 3-hydroxyacyl-CoA dehydrogenases could be distinguished (Clustal X, Thompson et al. [1997]). The first group comprises two well-studied enzymes from *E. coli*, *Pseudomonas mendocina* and mammalians (as an example, *Rattus norvegicus*) with the so-called *E. coli*-like structure of 3-hydroxyacyl-CoA dehydrogenases. The representatives of the second group with an inverted order of the domains are less studied and are referred here to as *Burkholderia*-like enzymes.

### Determination of the molecular mass

The apparent molecular weight of FadB’ with N-terminal His-tag defined by SDS-PAGE was 86 kDa, which corresponds to the theoretically calculated molecular weight of one subunit (see Additional file 1) (Laemmli [1970]; Weber and Osborn [1969]). The molecular weight of the native enzyme as determined by gel permeation chromatography (Superdex 200 HP) indicated a monomeric structure (87 kDa).

### Enzyme assays

(i) *Enoyl-CoA hydratase activity*. Specific activity of FadB’ with *trans*-crotonyl-CoA reached 98 ± 3 μmol mg^−1^ min^−1^. The intermediate and products of the reaction, 3-hydroxybutyryl-CoA and acetyl-CoA were detected via RP-HPLC/MS. Since no *cis*-crotonic acid was commercially available, it was not possible to measure the FadB’ activity with the CoA-thioester of this substrate.

**Figure 3:**
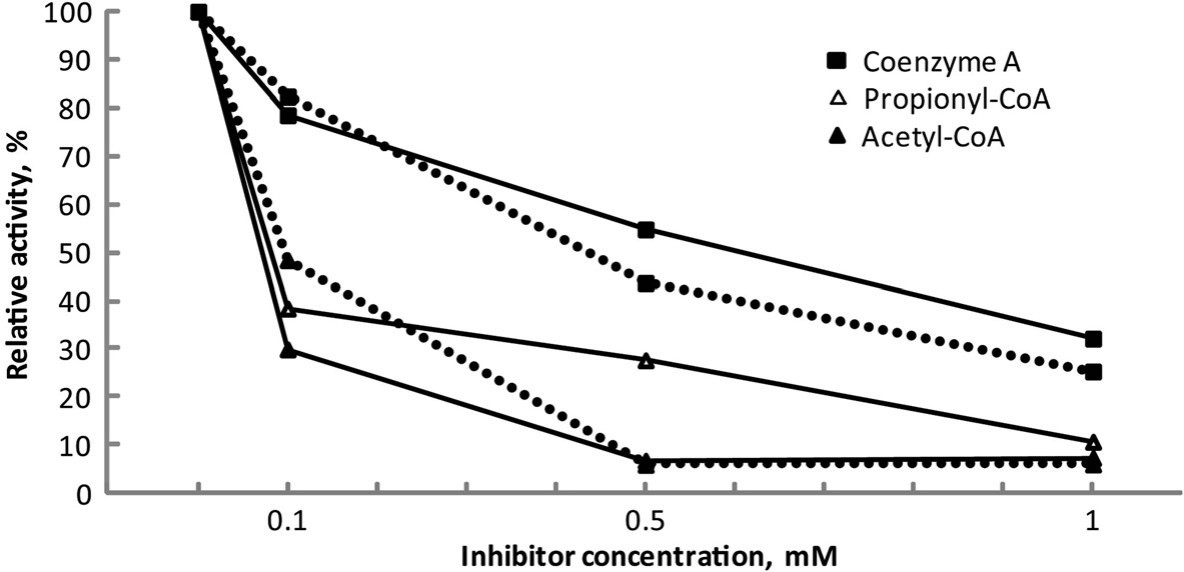
**Dependence of the inhibition of FadB’ activity on the concentration of acetyl-CoA, propionyl-CoA and CoA.** The dotted lines demonstrate the inhibitory effect on FadB’ activity measured with acetoacetyl-CoA as substrate. The reaction mixture contained in 100 mM Tris–HCl buffer (pH 7.0) 0.1 mM acetoacetyl-CoA, 0.2 mM NADH, 0.3 μg FadB’_*Re*_ and the inhibitor CoA or acetyl-CoA at the concentrations as indicated. The straight lines demonstrate the inhibitory effect on FadB’ measured with crotonyl-CoA as substrate. The reaction mixture contained in 100 mM Tris–HCl (pH 8.1) 0.1 mM crotonyl-CoA, 2 mM pyruvate, lactate dehydrogenase (9 U), 1.5 mM NAD^+^, 25 mM MgCl_2_ and the inhibitor CoA, acetyl-CoA or propionyl-CoA at the concentrations as indicated. All values are presented as mean values of at least two measurements, standard deviations varied between 0.01% and 3.58%.

Enoyl-CoA hydratase activity of FadB’ was inhibited by 70% in presence of 0.1 mM acetyl-CoA and by 30% in presence of 0.1 mM CoA (Figure 3). Propionyl-CoA was tested additionally to verify the inhibitory effect. Similarly, as with acetyl-CoA about 60% of the FadB’ activity was lost in presence of 0.1 mM propionyl-CoA. Higher concentrations of the mentioned CoA-thioesters and CoA inhibited the FadB’ activity stronger. 0.5 mM propionyl-CoA reduced the activity of FadB’ to 28% and 0.5 mM acetyl-CoA resulted in only 7% of residual activity. However, FadB’ retained 7% of the initial activity in presence of 1 mM acetyl-CoA and 11% - in presence of propionyl-CoA.

*(ii) 3-Hydroxyacyl-CoA dehydrogenase activity.* FadB’_*Re*_ showed maximal activity with NADH (92.7 ± 3.8 μmol mg^−1^ min^−1^) and was less active with NADPH (about 5 %, 4.9 ± 1.5 μmol mg^−1^ min^−1^) (Table 2). Kinetic values for acetoacetyl-CoA were determined. FadB’ exhibited a high affinity to acetoacetyl-CoA (*K*_*m*_ 48 μM) and a *V*_*max*_ of 149 μmol m ^-1^min^-1^. Presence of 0.1 mM acetyl-CoA resulted in the same *V*_*max*_ (149 μmol mg^-1^ min^-1^) values but a higher *K*_*m*_ (76 μM). The optimum pH was between 6 and 7, and the activity decreased with acidic and basic pH; at pH 5 and 8 the enzyme showed 65% and 75%, respectively, and at pH 10 only 46% of the maximal activity. An acidic pH of 4 gave only 26% of maximal activity. Dependence of FadB’ on the temperature revealed that no increase of specific activity was determined after 35°C and the highest activity was observed at 35 and 40°C. FadB’ lost 20% and almost 50% of its activity with acetoacetyl-CoA in presence of 0.1 mM CoA or 0.1 mM acetyl-CoA, respectively (Figure 3). Further increases of the inhibitor concentrations reduced the activity of FadB’ drastically.

**Table 2:** Enzyme characteristic of FadB’ in comparison with other enzymes

	**Cofactor**		**Trifunctional activity**	**Specificity to (*S*)- or (*R*)-3-hydroxyacyl-CoA**	**Reference**
	**NAD**^**+**^	**NADP**^**+**^			
FadB’_*Re*_	100^*^	5^*^	+	S^**^	this work
PhaB1_*Re*_	20	100	-	R	Haywood et al. [1988b]
Unidentified acetoacetyl-CoA reductase (*R. eutropha* H16)	100	2	n.a.	S/R	Haywood et al. [1988b]
FadB_*Ec*_	+	n.a.	+	S	Binstock and Schulz [1981]

*-The activity of FadB’ was measured at 340 nm and 30°C with acetoacetyl-CoA in direction of 3-hydroxybutyryl-CoA formation as described in “Materials and Methods”.

**-The stereospecificity of FadB’ was measured at 303 nm and 30°C with (*S*)- and (*R*)-3hydroxybutyryl-CoA as described in “Materials and Methods”.

*(iii) Stereospecificity of 3-hydroxyacyl-CoA dehydrogenase*. To analyze the stereospecificity of FadB’ the formation of (*S*)- or (*R*)-3-hydroxybutyryl-CoA from (*S*)- or (*R*)-3HB and acetyl-CoA was carried out by an auxiliary enzyme Pct *in situ* in a similar way as described before (Volodina et al. [2014]). FadB’_*Re*_ converted (*S*)-3-hydroxybutyryl-CoA to acetoacetyl-CoA (16.5 ± 0.4 μmol mg^−1^ min^−1^), while no conversion of (*R*)-3-hydroxybutyryl-CoA was detected. Formation of (*R*)-3-hydroxybutytryl-CoA by Pct was confirmed by employing RP-HPLC/MS. Relative activity of FadB’ measured with NADP^+^ was less than 10% in comparison to the activity measured with NAD^+^ (1.5 ± 0.3 μmol mg^−1^ min^−1^).

## Discussion

The expression of the β-oxidation genes in *R. eutropha* H16 is up-regulated, when the cells are grown in presence of FAs (Brigham et al. [2010]). Two operons located on chromosome #1 are responsible for the degradation of FAs in *R. eutropha* H16: H16_A0459-A0464 and H16_A1526-A1531. The latter operon contains among others the gene for a 3-hydroxyacyl-CoA dehydrogenase/enoyl-CoA hydratase (FadB1), which is similar to the enzymes from *E. coli* (Insomphun et al. [2014]). On the other hand, FadB’_*Re*_ from H16_A0459-A0464 due to its primary structure belongs to the *Burkholderia*-like subgroup of 3-hydroxyacyl-CoA dehydrogenases (Figure 2). It exhibits an inverted order of catalytic domains in comparison to FadB1, well-studied enzymes from mammalians and *E. coli*. FadB’ is more related to the enzymes from *B. subtilis* and *C. vinosum*. Apparently FadB’ and FadB1 have different ancestors and fusion of both 3HCDH_N and ECH domains in FadB’ and FadB1 has occurred independently and might be the result of a convergent evolution. In addition, the separate enoyl-CoA hydratase is located on the same operon downstream of the *fadB’*. Nevertheless, it has been shown that the enoyl-CoA hydratase domain of FadB’ retains its activity. Since both genes (H16_A0464, enoyl-CoA hydratase and H16_A0461, FadB’) were found to be expressed in presence of fatty acids (Brigham et al. [2010]), they seem to function simultaneously and might exhibit different substrate specificities.

Thus, FadB’ is the first enzyme of the fatty acid metabolism of *R. eutropha* characterized *in vitro*. Heterologously expressed His-tagged FadB’ exhibited a single monomeric structure. However, an oligomeric structure of the enzyme *in vivo* cannot be excluded, as the enzyme might function as part of a multienzyme complex, similarly as FadB in *E. coli*. Nevertheless the solely expressed and folded FadB’_*Re*_ was active, both in the absence of potential co-monomer(s), such as β-ketothiolase, and in presence of N-terminal His-tag. Since, the activities for both, the enoyl-CoA hydratase and 3-hydroxyacyl-CoA dehydrogenase reactions, were determined, the mechanism of the reaction is presumably as follows: enoyl-CoA, once being captured by FadB’, undergoes two consequent conversions finally yielding 3-ketoacyl-CoA (Figure 1).

FadB’ was found to be an NADH-dependent enzyme. 3-Hydroxyacyl-CoA dehydrogenases from β-oxidation are presupposed to utilize NADH as a cofactor, due to the organisation of the central metabolism. One cycle of β-oxidation reduces one NADH molecule and produces one molecule of acetyl-CoA. The latter is directed to the Krebs cycle, which supplies three NADH molecules pro cycle. On the other hand, NADP(H) could also be used as a cofactor, although the enzyme showed only 5-10% of the activity with NADP(H) in comparison to NAD(H). However, NADPH is the main cofactor of PHB synthesis and FA *de novo* synthesis and, consequently, FadB’ from FA degradation favours NAD(H), avoiding a competition for the cofactor with other enzymes. FadB’ was found to be strictly (*S*)-stereospecific with 3-hydroxybutyryl-CoA, which is also characteristic for enzymes from β-oxidation. This corroborates with the theory, that the fatty acid metabolism does not intersect the PHB synthesis and (*R*)-isomers are the only precursors of PHB synthase, and (*S*)-isomers are engaged in the degradation of fatty acids. Thus, as expected, FadB’ does not compete with PhaB from PHB metabolism for the substrates. Nevertheless, the deletion of the FadB’ encoding gene led to a decrease of PHA content, suggesting an indirect role of FadB’ in PHA precursor supply (Insomphun et al. [2014]).

Interestingly, acetyl-CoA and propionyl-CoA, which are, on one hand, the end products of FA degradation and, on the other hand, the precursors for the PHA synthesis, are inhibiting FadB’. The activity of FadB’ in presence of free CoA was also lower (Figure 3). The inhibitory effect of high concentrations of these substrates might demonstrate a modulatory character of the FA degradation pathway. The end products of β-oxidation seem to negatively affect the rate of FA oxidation in *R. eutropha*. Additionally, free CoA, elaborated in Krebs cycle, is known to inhibit the β-ketothiolase from PHB synthesis (Oeding and Schlegel [1973]). So, during the growth phase abundant acetyl-CoA is oxidized releasing CoA, and PHB synthesis is slowed down. Apparently the activity of at least one enzyme from FA degradation, FadB’, is inhibited by the increased concentration of CoA, acetyl-CoA and propionyl-CoA. Concerning the mechanism of the inhibition, it was shown, that with increasing concentrations of acetoacetyl-CoA the inhibitory effect of acetyl-CoA was weakened. A higher apparent *K*_*m*_ value and unchanged *V*_*max*_ for acetoacetyl-CoA in presence of acetyl-CoA suggest competitive mechanism of acetyl-CoA’s inhibition; however, double reciprocal Lineweaver-Burk analysis (data not shown) revealed a mixed type of inhibition. Recently, it was demonstrated that if only FadB’ was active in *R. eutropha* lacking *fadB1*, the mutant stored comparable amounts of PHA as the wild type but with higher 3HHx content (Insomphun et al. [2014]). Taken into a consideration, that during degradation of long chain fatty acids, the concentration of acetyl-CoA/propionyl-CoA increases and the relative concentration of long chain fatty acids decreases, this might cause the inhibitory effect on FadB’. Under these conditions in the strain lacking *fadB1* the FAs are, putatively, not degraded completely and more 3HHx is available for the PHA synthesis. The regulation of FA degradation seems to be linked between the PHA synthesis and the central metabolism. First, the deletion of FadB’ has an indirect influence on PHA synthesis, when the strain is grown on fatty acids (Insomphun et al. [2014]). Second, the activity of FadB’ is inhibited by high concentrations of the end products of FA degradation and Krebs cycle.

The existence of two analogous enzymes, FadB’ and FadB1, demonstrates genetic diversity of *R. eutropha.* The presence of multiple isoenzymes is beneficial for the organism for an adaptation to changing environments and survival. As it was shown, although both *fad* operons function simultaneously, deletion of only one of them does not impair the fatty acid utilization capacity of *R. eutropha* (Brigham et al. [2010]). Multiple copies of β-ketothiolases and acetoacetyl-CoA reductases assist in PHB metabolism and compensate the lack of one or several genes (Budde et al. [2010]; Lindenkamp et al. [2012]).

The determination of the substrate specificity of FadB’ with medium and long chain length acyl-CoAs especially in comparison to FadB1 and additional enoyl-CoA hydratase (H16_A0464) could provide in the future more information about the role of FadB’ in β-oxidation. 3-Hydroxyacyl-CoA dehydrogenase/enoyl-CoA hydratase from *E. coli*, for example, possesses also Δ^3^-*cis*,Δ^2−^*trans*-enoyl-CoA isomerase and (*S*)/(*R*)-epimerase activities. However, further studies have to be done to clarify, if FadB’ from *R. eutropha* despite its different domain organisation is also capable of converting *cis*- and (*R*)-isomers of CoA thioester to the *trans*- and (*S*)-stereoisomers, respectively.

Thus, FadB’ as well as 3-hydroxyacyl-CoA dehydrogenases (*paaH1* and *paaH2*) and acetoacetyl-CoA reductases (*phaB1* and *phaB3*) are able to convert 3-ketoacyl-CoAs to 3-hydroxyacyl-CoAs (Table 2; Budde et al. [2010]) and, therefore, are interesting for biotechnical applications. It is assumed that (*S*)-3-hydroxyacyl-CoA dehydrogenase (PaaH1) might refer to the non-stereospecific NADH-dependent acetoacetyl-CoA reductase with broad substrate range purified by Haywood et al. ([1988b]) (Machado et al. [2012]), however, a mixture of the above mentioned enzymes could have been purified. PhaB1 and PhaB3 are important for PHA synthesis. Though, additional application of (*S*)-specific FadB’ together with PhaB can enhance the flux of the β-oxidation intermediates to PHA synthesis during growth on FAs. PaaH1 has been applied in an artificial alcohol synthesis pathway in *E. coli* (Machado et al. [2012]). This pathway comprises, amongst others, three reverse steps of β-oxidation, catalyzed by three separate enzymes: β-ketothiolase, 3-hydroxyacyl-CoA dehydrogenase and enoyl-CoA hydratase reactions. Concerning that FadB’ assisted in the incorporation of 3HHx into a copolymer (Insomphun et al. [2014]) and exhibited the enoyl-CoA hydratase activity, it might be promising to exchange the PaaH1 and a crotonase with FadB’ in linear alcohol synthesis pathway. (*S*)-Stereospecific 3-hydroxyacyl-CoA dehydrogenases are also interesting for biotechnological production of enantiomeric pure compounds *in vivo* (Lee et al. [2008]). Since FadB’ can be purified in one step and, if stored in glycerol, it does not loose activity for months, making the FadB’ applicable as a coupling enzyme for *in vitro* assays (Volodina et al. [2014]).

## Competing interests

The authors declare that they have no competing interests.

## Authors’ contributions

EV conceived, designed and performed the experiments. Acquisition of data, analysis and interpretation of data: EV. AS supervised the work and has been involved in drafting the manuscript, revising it critically for important intellectual content; and has given final approval of the version to be published. Both authors read and approved the final manuscript.

## Additional file

## Supplementary Material

Additional file 1: SDS‐PAGE of FadB’ after His‐Tag purification. Click here for file
