# Supplementary figures and images for: (S)-3-hydroxyacyl-CoA dehydrogenase/enoyl-CoA hydratase (FadB’) from fatty acid degradation operon of Ralstonia eutropha H16
Source: AMB Express. 2014 Aug 28;4:69. doi: 10.1186/s13568-014-0069-0 (PMC4230905; doi:10.1186/s13568-014-0069-0)

**Additional file 1** SDS-PAGE of FadB' after His-Tag purification.

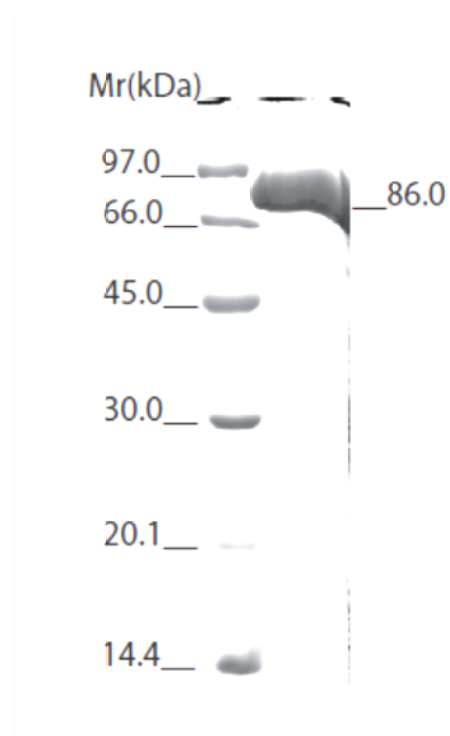

Supplement: Additional file 1: — SDS‐PAGE of FadB’ after His‐Tag purification. [file s13568-014-0069-0-S1.pdf]
